# Novel insights on association and reactivity of Bispectral Index, frontal electromyogram, and autonomic responses in nociception-sedation monitoring of critical care patients

**DOI:** 10.1186/s12871-022-01864-6

**Published:** 2022-11-15

**Authors:** Juhani A. Stewart, Mika O. K. Särkelä, Johanna Wennervirta, Anne P. Vakkuri

**Affiliations:** 1 Cardiology, Faculty of Medicine, University of Helsinki and Helsinki University Hospital, Stenbäckinkatu 9, PL 100, 00029 HUS Helsinki, Finland; 2 GE Healthcare Finland Oy, Helsinki, Finland; 3 Department of Anesthesiology and Intensive Care, Helsinki University Hospital, Helsinki, Finland

**Keywords:** Bispectral Index, Blood pressure variability, Electroencephalogram, Neuromonitoring, Responsiveness Index, Intensive care

## Abstract

**Background:**

Assessing nociception and sedation in mechanically ventilated patients in the ICU is challenging, with few reliable methods available for continuous monitoring. Measurable cardiovascular and neurophysiological signals, such as frontal EEG, frontal EMG, heart rate, and blood pressure, have potential in sedation and nociception monitoring. The hypothesis of this explorative study is that derived variables from the aforementioned signals predict the level of sedation, as described by the Richmond Agitation-Sedation score (RASS), and respond to painful stimuli during critical care.

**Methods:**

Thirty adult postoperative ICU patients on mechanical ventilation and receiving intravenous sedation, excluding patients with primary neurological disorders, head injury, or need for continuous neuromuscular blockage. Bispectral Index (BIS), EMG power (EMG), EMG-derived Responsiveness Index (RI), and averaged blood pressure variability (ARV) were tested against RASS measurements. The aforementioned variables together with blood pressure and Surgical Pleth Index (SPI) were explored before and after painful stimuli (for example bronchoscopy, or pleural puncture) at varying RASS levels, to test variable responsiveness.

**Results:**

BIS, EMG, and RI predicted RASS levels with a prediction probability (P_K_) of 0.776 for BIS, 0.761 for EMG, and 0.763 for RI. In addition, BIS, EMG, and ARV demonstrated responsiveness to painful stimuli during deep sedation (RASS score ≤ -3).

**Conclusion:**

Variables derived from EEG and EMG are associated with sedation levels, as described by the RASS score. Furthermore, these variables, along with ARV, react with consistency to painful stimuli during deep sedation (RASS -5 to -3), offering novel tools for nociception-sedation monitoring of mechanically ventilated ICU patients requiring deep sedation.

## Introduction

Sedation and analgesia are a crucial part of critical care but optimizing these in non-communicative patients can be challenging. Deep sedation is common, with a prevalence of 35% to 68% in mechanically ventilated patients, and excessive sedation is associated with adverse outcomes, such as a higher mortality and longer ICU stays [1–5]. Several randomized studies have shown improved outcomes with strategies avoiding over-sedation, however insufficient sedation increases both patient agitation and staff work load, and may compromise patient safety [2, 6].

One of the main challenges in detecting and treating pain and stress in ICU patients is the lack of suitable monitors of nociception and analgesia [1, 2, 7, 8]. Assessing abstract concepts such as pain and suffering in patients unable to self-report (i.e. measuring nociception) is typically based on observing behavioural and autonomic physiological responses. Of these, the latter might provide an objective monitoring medium [7–10], however, the basic physiological parameters (such as heart rate and blood pressure) alone are not accurate enough for pain assessment [9, 11].

Derived frontal electroencephalogram (EEG) and electromyogram (EMG) variables can be used as noninvasive neuromonitoring methods of sedation and anesthesia depth. The most widely used EEG derived variable is the Bispectral Index (BIS), which has been validated for perioperative sedation [12] and has showed positive results in monitoring ICU sedation [2, 5, 6, 9, 13, 14]. The increase of BIS during tracheal suction can be alleviated by premedication with alfentanil according to Brocas et al*.*, implying a potential use of BIS for nociception-analgesia monitoring [10]. The Responsiveness Index (RI) is an EMG-derived variable, proposed for sedation monitoring in the ICU. To determine RI the frontal EMG is measured with a forehead sensor, EMG power is derived from each 0.5 s epoch, and finally RI is derived based on the EMG power time series of the last 60 min [15]. Both BIS and RI provide real-time monitoring with a simple scale from 0 to 100, with low values representing deep sedation and higher values representing increasing arousal [12, 15–19].

The physiological stress responses to pain (tachycardia, hypertension, diaphoresis) can be blunted in ICU patients, mainly due to medication (sedatives, analgesics, muscle relaxants, blood pressure medication) [7, 9]. The forehead muscles are less sensitive to neuromuscular blockade agents (NMBAs) [20], and frontal EMG reactivity to nociception should remain [20] even after classical signs (such as tachycardia and hypertension) are absent due to medication [7, 21–23].

Short-term blood pressure variability (BPV) is an interesting variable for assessing nociception and analgesia, as the autonomic responses of heart rate and blood pressure are inherently linked to each other [9, 11, 23, 24]. The variability of heart rate and blood pressure, along with direct increases in heart rate and systolic blood pressure, have been linked to nociception [9, 22, 25].

The Surgical Pleth Index (SPI), a derived variable combining normalized pulse photoplethysmographic waveform amplitude (PPGA) and RR interval (RRI), monitors nociception by reflecting the changes in the balance of sympathetic and parasympathetic tone [8, 9, 23]. Extensive studies have evaluated the use of SPI in surgical anaesthesia, but published studies of use in critical care are lacking [8].

The aims of this prospective and explorative study were to test the performance of sedation monitoring variables derived from EEG, EMG and hemodynamic measurements (heart rate, blood pressure, BPV, SPI), against Richmond Agitation-Sedation scores (RASS), and their responsiveness to painful stimuli during critical care.

## Methods

The inclusion criteria for this study were adult patients, with a planned or unplanned postoperative admission to the ICU, on mechanical ventilation via an endotracheal tube, and receiving continuous intravenous sedation (propofol, midazolam). Exclusion criteria were primary neurological disorders (including stroke, probable hypoxic brain injury, intracranial hemorrhage, and head injury with reduced level of consciousness), the continuous use of NMBAs during monitoring, confirmed central nervous system infection, or a short data collection time (less than 12 h). Sparing bolus administration of NMBAs to facilitate ventilation were allowed, as frontal EMG is reasonably resistant to the effects of partial blockade.

Patient recruitment and data collection took place from the 7^th^ of May 2007 to the 1^st^ of April 2009, with dedicated study nurses gathering all data. Of the 32 recruited patients, 30 were included (2 excluded due to short data collection time). The study period was from arrival to the ICU until extubation, with continuous monitoring (EEG, EMG, hemodynamic parameters). During daytime one of two dedicated research nurses conducted computerized and standardized RASS assessments every 60 min (based on the RASS score), with increasing stimuli given every minute (first a 90 dB verbal command from headphones, followed by 105 dB white noise from headphones, followed by a peripheral train-of-four nerve stimulation, followed by peripheral nerve tetanic stimulation). All observed painful stimuli which were estimated to be significantly painful, and which require at least local anaesthesia, were documented (including bronchoscopy, at least 18 G/5.4 mm diameter chest tube insertion for pleural drainage, tracheal airway suction [10], and train-of-four/tetanic nerve stimulations). All given medications and patient reactions to the previously specified painful stimuli were annotated. Data on the cumulative dose of sedative drugs (including propofol and midazolam), opioid analgesia (including fentanyl, oxycodone, sufentanil and buprenorphine), and muscle relaxants were recorded for the whole monitoring period. During mechanical ventilation, a target RASS of -2 to 0 was used as a sedation guideline.

### Monitoring methods

A BIS sensor was positioned in the standard position on the patient’s forehead, from which BIS and EMG values were monitored with the E-BIS module of GE Datex-Ohmeda S/5 monitoring system (BIS XP, algorithm version 4.0, smoothing rate 15 s). The Entropy sensor was positioned bilaterally on the forehead, above the BIS sensor [15]. The RI values were retrospectively calculated from the EEG/EMG signal obtained from the Entropy sensor and E-Entropy module (GE Healthcare, Helsinki, Finland). Quality of BIS and RI signals were controlled with automatic sensor checks, and both sensors were changed every 24 h. Plethysmographic pulse waveform signal was acquired from the GE SpO_2_ sensor and measurement module. SPI, and its subcomponents PPGA and RRI, were derived offline using the plethysmographic pulse waveform signal and the SPI program code of GE Carescape monitor (GE Healthcare, Helsinki, Finland). Invasive blood pressure data, including systolic (sysBP) and diastolic (diaBP) blood pressure, were monitored from a peripheral arterial line. Mean values of sysBP and diaBP were stored at 10 s intervals. The sysBP time series were used to derive average real variability (ARV) [26], a mathematical variable describing BPV:

$${ARV(sysBP)}_m=\frac1{N-1}{\textstyle\sum_{k=m-N}^{m-1}}\left|{sysBP}_{k+1}-{sysBP}_k\right|,$$

where *N* was 180, i.e., a 30-min time-window was used. SysBP measurements greater than 280 mmHg or lower than 50 mmHg and an increase of over 100 mmHg in 10 s were discarded as artifacts.

All patients were monitored continuously with 3-lead ECG and a peripheral arterial line with invasive blood pressure monitoring, and all ECG results were reviewed offline by a cardiologist (J.S.). Monitoring data were captured with the S/5 Collect SW (GE Healthcare, Helsinki, Finland).

### Statistical methods

In this prospective and exploratory study, variables for RASS comparison were selected from variables used to monitor the depth of anesthesia or sedation (i.e., BIS, EMG, and RI). As the preliminary visual analysis suggested a close resemblance between RI and ARV, ARV results were compared against RASS too. Number of study patients shown in high case (*N*), while the number of measurements are shown in low case (*n*). Blood pressure and heart rate by themselves are not predictive of the level of sedation or nociception [9, 11], but the variability of these potentially might be, and were therefore considered for further analysis. Heart rate variability (HVR) is strongly affected by non-sinus rhythms, which is common and represented a fifth of the data of this study, and therefore heart rate and variability were not submitted for further analysis.

Associations of BIS, EMG, RI and ARV to RASS levels were analysed with prediction probability (P_K_). P_K_ is an established statistical method for quantifying the ability of an anesthetic depth indicator to decrease consistently with deepening anesthesia. Although it is more commonly used for binary categories, such as comparing the variable value of the responsive state against its value in a non-responsive state, the methodology can be similarly applied to more than two-ordered categories [27]. In our material, a P_K_ of 1 would indicate that the variable value will monotonically decrease with deepening sedation from RASS + 2 to RASS -5, whereas a P_K_ of 0.5 would indicate that the variable’s capability for predicting RASS is equal to flipping a coin. As the data contained multiple samples from each patient, with different amounts of samples for each patient, we used the stratified bootstrapping method as proposed by Lüginbuhl et al. [28]. The method randomly selects one sample from every patient and derives the P_K_ value using the jack-knife method for this subset of data (in this case 30 samples) [27]. This procedure is repeated 1000 times, and the presented P_K_ values are the medians of the 1000 subset P_K_ values. The value of each variable was recorded just prior to the start of each RASS assessment, so that the stimulus of the assessment itself does not affect the recorded variables.

To evaluate EMG influence on BIS and RI, we divided all RASS observations to low and high EMG groups according to a threshold value of 30 dB [29]. To evaluate the possible influence of the autonomous nervous system on BIS and RI, we divided all RASS observations into two equal sized groups by the median ARV value. In both analyses’, we derived P_K_ values separately for each group with the presented random subsampling method.

We were further interested in studying the responsiveness of the variables to painful stimuli at different RASS levels. For this analysis, we selected the mean value of each variable from a time period 2 to 5 min prior to each registered stimulus, and the mean value from a time period of 0 to 3 min after each stimulus. Successive stimuli occurring within 10 min of the earlier stimulus were not included in the analysis. For the analysis we selected all the variables used in the study, including systolic and diastolic blood pressures, SPI and SPI subcomponents of RRI and PPGA. Wilcoxon signed rank test with Bonferroni correction was applied to study whether pre and post stimuli values were from the same population, the type I error was set at 5% (two-sided) which resulted in a Bonferroni corrected limit of statistical significance at α = 0.0056.

All statistical analyses were performed with Matlab 9.5 (The MathWorks Inc., Natick, MA, USA).

## Results

All demographic data and data from the ICU treatment period are presented in Table 1, with a study period of approximately 1.5 to 3.0 days beginning from ICU admission. Of all the monitored ECG data 19% were non-sinus rhythm (e.g. atrial fibrillation, atrial flutter, or pacemaker rhythm). To facilitate ventilation, bolus NMBAs were administered sparingly in 12 (40%) patients. Of these, 11 patients received rocuronium with a cumulative median (range) dose of 60 mg (30–450 mg), and 1 patient received cisatracurium with a cumulative dose of 14 mg. The patient with the highest dose of NMBAs (rocuronium 450 mg) had pulmonary hypertension and was treated in the ICU with nitrous oxide inhalation for acute respiratory distress syndrome after cardiac surgery.

**Table 1:** Demographic and clinical data of all study patients (*N* = 30). The study monitoring time begins from admission to ICU. Values are given as median (range), or total number of patients (%, percentage of all patients in subgroup), as appropriate

Parameter	Value
Age (years)	59 (30 to 80)
Gender, female/male (*N*, %)	12 (40%) / 18 (60%)
BMI (kg/m^2^)	27.8 (23.7 to 33.5)
Study monitoring time (h)	50 (31 to 70)
Propofol infusion dose during monitoring period (mg/kg/h)	1.2 (0.0 to 3.9)
Hourly opioid dose as morphine equivalent (mg/h)	1.4 (0.1 to 33.3)
Emergency ICU admittance	6 (20%)
Planned postoperative ICU admittance	18 (60%)
ICU LOS (days)	17 (2 to 37)
Hospital LOS (days)	18 (2 to 42)
SOFA score on 1st day	8 (4 to 15)
Discharged to a high-dependency unit	4 (14%)
In-hospital death	4 (13%)
Main reason for admittance to ICU:	
*Gastro-intestinal*	8 (27%)
*Cardiac*	6 (20%)
*Pancreatitis*	5 (17%)
*Ruptured abdominal aortic aneurysm*	4 (13%)
*Infection*	4 (13%)
*Urologic*	1 (3%)
*Thoracic*	2 (7%)
Electrocardiogram dominant rhythm:	
*Sinus rhythm*	81%
*Atrial fibrillation*	12%
*Pacemaker rhythm*	7%

*Abbreviations: BMI* Body mass index, *ICU* Intensive care unit, *LOS* Length of stay, *SOFA* Sequential organ failure assessment

### Level of sedation

Table 2 presents the results of P_K_ analysis of 406 pairs of RASS score and variable values (for RASS distribution, see Table 5). Of the tested variables, BIS, RI and EMG demonstrated a moderate association with RASS.

**Table 2:** Prediction probabilities (P_K_) for monitored parameters, compared against the Richmond Agitation-Sedation Score (RASS). P_K_ was estimated from 1000 jack-knife samples, each including one parameter-RASS observation pair from each patient (*N* = 30). The table presents the median P_K_ of 1000 subset P_K_ values, with interquartile range (IQR). A total of 406 RASS assessments were available for analysis

Parameter	P_K_ [IQR]
Bispectral Index (BIS)	0.776 [0.739, 0.808]
Frontal electromyogram power (EMG)	0.761 [0.719, 0.795]
Responsiveness Index (RI)	0.763 [0.728, 0.799]
Blood pressure average real variability (ARV)	0.549 [0.504, 0.596]

All the P_K_ values for BIS and RI in both low and high EMG groups, and low and high ARV groups, are presented in Table 3. The BIS P_K_ values tend to be lower with a low EMG activity, whereas RI P_K_ values decrease with high EMG activity. Within the ARV subgroup analyses, BIS had similar P_K_ values in both high and low ARV groups, whereas RI demonstrated lower P_K_ values in the low ARV subgroup.

**Table 3:** Presenting the medians and interquartile ranges (IQR) of jack-knife prediction probabilities (P_K_) for Bispectral Index (BIS) and Responsiveness Index (RI), compared against the Richmond Agitation-Sedation Score (RASS). Comparisons are grouped into low and high EMG groups (upper part of table), and into low and high blood pressure averaged real variability (ARV) groups (lower part of table). A total of 1000 jack-knife P_K_ estimates were derived, each estimate including one sample of *N* patients in the group

	Low EMG	High EMG
Parameter	*N* = 24	*N* = 29
Bispectral Index (BIS)	0.716 [0.684, 0.749]	0.736 [0.695, 0.777]
Responsiveness Index (RI)	0.749 [0.718, 0.775]	0.716 [0.673, 0.755]
	Low ARV	High ARV
Parameter	*N* = 28	*N* = 27
Bispectral Index (BIS)	0.770 [0.736, 0.800]	0.789 [0.756, 0.821]
Responsiveness Index (RI)	0.723 [0.685, 0.761]	0.770 [0.742, 0.801]

**Fig. 1:**
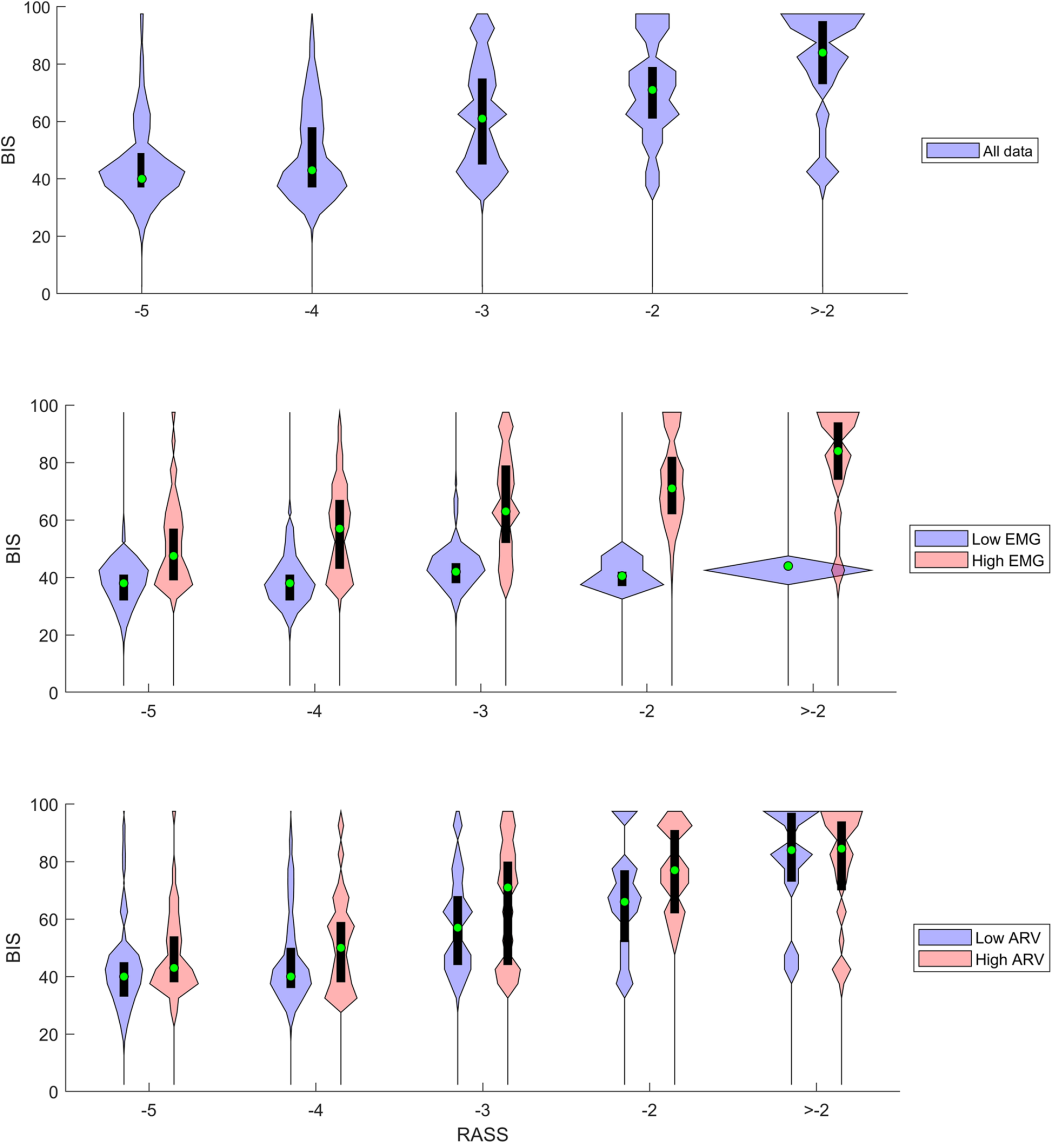
Violin plot diagrams (black boxes for quartiles, green circles for median values, envelopes presenting distribution) of the BIS values at different RASS scale levels, with the first figure presenting the results of all data, the second figure presenting the data divided into low EMG (cyan) and high EMG (pink) groups, and the third figure presenting the data divided into low ARV (cyan) and high ARV (pink) groups. Median BIS increases with the increasing RASS values, and this effect is dependent of the EMG power. With a high EMG power the BIS-RASS correlation is evident, while in the low EMG power group BIS does not correlate with the RASS levels

**Fig. 2:**
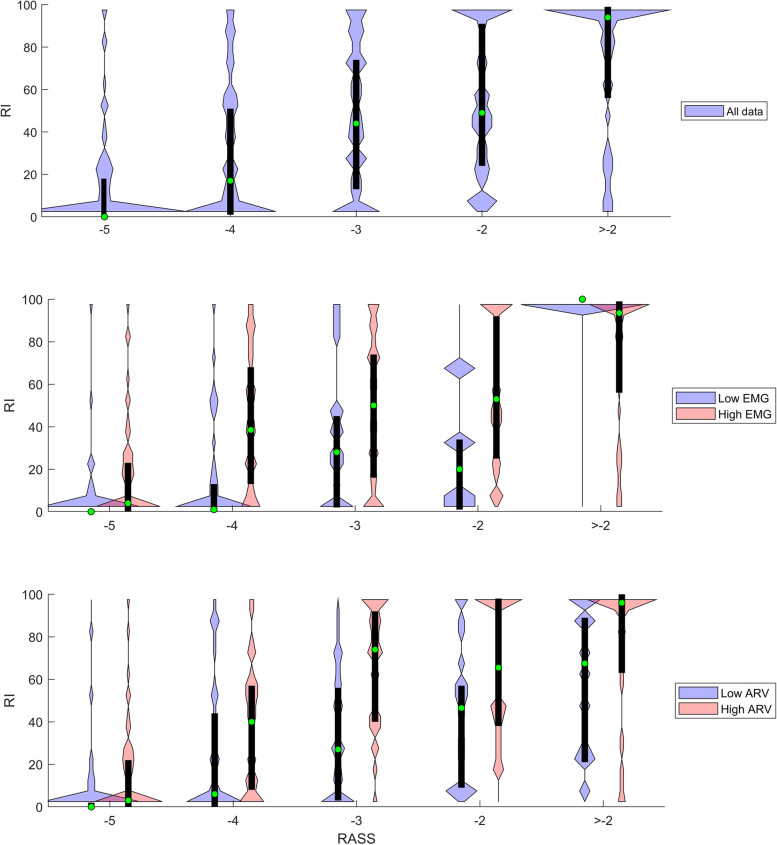
Violin plot diagrams (black boxes for quartiles, green circles for median values, envelopes presenting distribution) of the RI values at different RASS scale levels, with the first figure presenting the results of all data, the second figure presenting the data divided into low EMG (cyan) and high EMG (pink) groups, and the third figure presenting the data divided into low ARV (cyan) and high ARV (pink) groups. Median RI increases with the increasing RASS values, and in the low ARV group the RI values tend to be lower than in the high ARV group

Figure 1 presents violin plot diagrams of BIS versus RASS, first using the whole data set, then grouped into both low and high EMG groups, and low and high ARV groups. From these groupings it is possible to see that the range of BIS values is mostly restricted to a range of 30 to 50 when EMG activity is low, but the ARV grouping into high and low does not have an effect on the BIS versus RASS association. Figure 2 presents violin plot diagrams of RI versus RASS for the whole data set and grouped into both low and high EMG groups, and low and high ARV groups. The RI values switch between two values (0 and 100), mainly when moving from RASS level -3 to -2 (decreasing sedation). This bimodal switching seems to be linked to the level of sympathetic activity, as with high ARV values the switch happens between RASS levels of -4 and -3, whereas with low ARV values the switch only occurs when RASS is above -2.

### Responsiveness to nociceptive stimuli

**Fig. 3:**
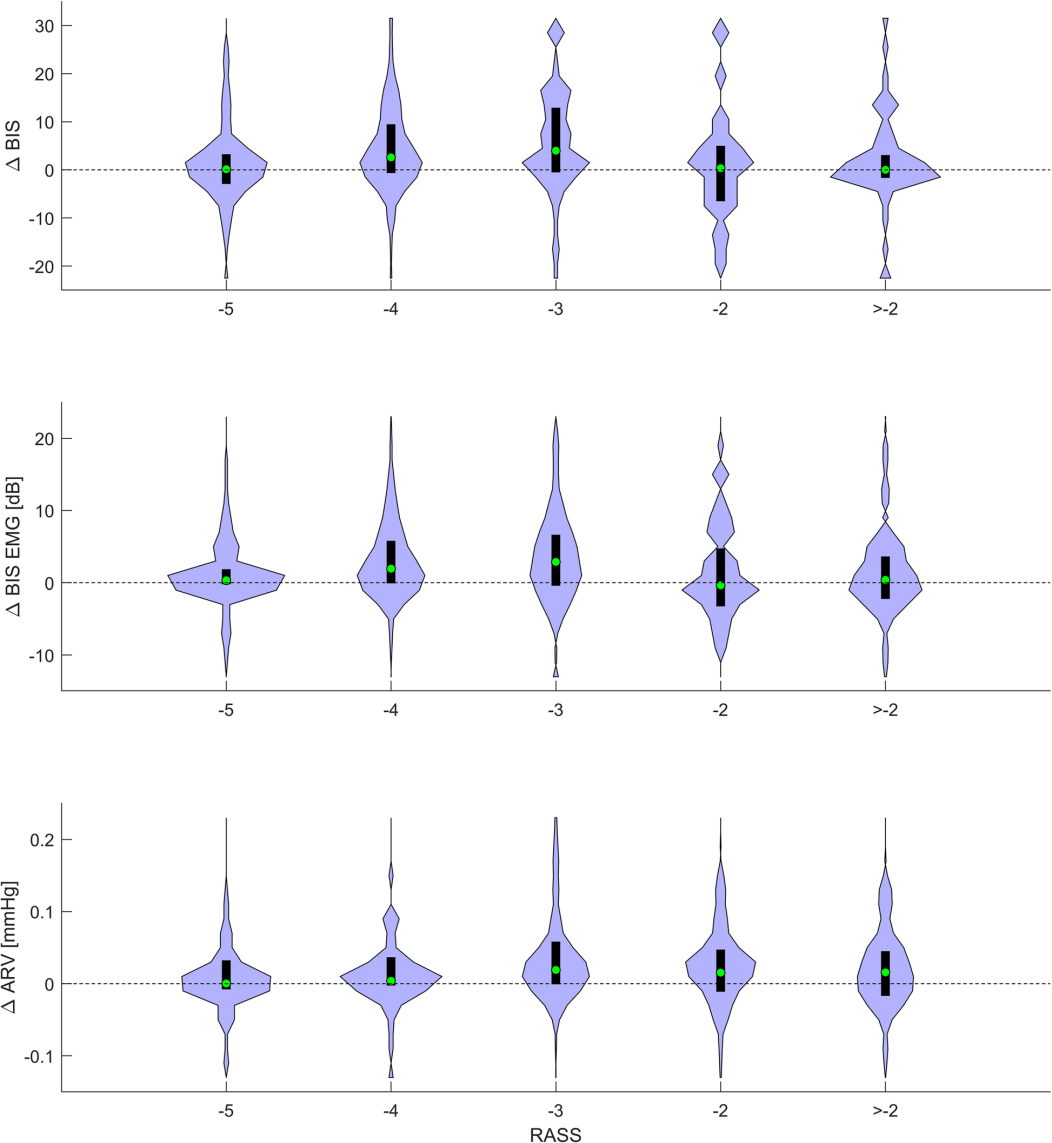
Violin plot diagrams (black boxes for quartiles, green circles for median values, envelopes presenting distribution) of the BIS, EMG and ARV responses to stressful stimuli at varying RASS levels. The ∆ values are derived by subtracting the prestimulus value from the poststimulus value

In Table 4 are presented the Wilcoxon signed rank test results of 524 stimulus–response pair analyses (for RASS distribution, see Table 5). Based on the results, the variables with significant *p*-values with two or more RASS levels were selected for further analysis. Such variables were BIS, EMG, and ARV. The differences between post and pre stimulus variable values (∆ BIS, ∆ EMG, ∆ ARV) at different RASS levels are presented in Fig. 3. All three variables responded most consistently at RASS levels -4 and -3, showing an increase in variable value in roughly 75% of the events.

**Table 4:** Responses of the tested parameters to the nociceptive stimuli (including the standardized study protocol train-of-four and tetanic testing, and painful procedures of standard care) at different Richmond Agitation-Sedation score (RASS) levels (*n* samples), analysed with the Wilcoxon signed rank test with Bonferroni correction (corrected α = 0.0056). A statistically significant *p* value indicates a consistent change after the stimulus (either decrease or increase) and is marked with an asterisk (*)

	RASS				
	-5	-4	-3	-2	> -2
Parameter	*n* = 141	*n* = 163	*n* = 98	*n* = 60	*n* = 63
Bispectral Index (BIS)	0.5547	< 0.0001*	< 0.0001*	0.9069	0.3944
Frontal electromyogram power (EMG)	< 0.0001*	< 0.0001*	< 0.0001*	0.4866	0.3109
Responsiveness Index (RI)	0.4158	0.0339	< 0.0001*	0.0779	0.0587
Systolic blood pressure	0.2339	0.0673	0.0258	0.4223	0.2725
Diastolic blood pressure	0.1248	0.0177	0.0016*	0.1532	0.0827
Blood pressure average real variability (ARV)	0.4278	0.0006*	< 0.0001*	0.0059	0.0178
Surgical Pleth Index (SPI)	0.0696	0.0051*	0.9157	0.2226	0.5407
RR Interval (RRI)	0.9108	0.0001*	0.2602	0.3588	0.8214
Plethysmograph amplitude (PPGA)	0.0121	0.0906	0.8456	0.7000	0.1644

**Table 5:** Presenting the *n* for all the different analysed pairs at different RASS levels. In the first group (Table 2) are the pairs of RASS and the analysed variables from before the RASS assessment presented in Table 2, including Bispectral Index (BIS), frontal EMG, Responsiveness Index (RI), and Averaged Blood pressure Variability (ARV). In the second group (Table 3) are the BIS/RI and RASS pairs presented in Table 3, grouped into low and high EMG and ARV groups. In the final group (Table 4) are the stimulus–response pairs presented in Table 4, which represent the pairing of a painful stimulus and measured variable values following stimulation. For the Table 4 Stimulus–response pairs, RASS ≥ -2 were pooled**

	Pairs	RASS (*n*)							
		-5	-4	-3	-2	-1	0	+ 1	+ 2
Table 2	Variable-RASS	104	100	98	51	35	11	6	1
Table 3	Low EMG power BIS/RI-RASS	52	46	19	4	1	-	-	-
	High EMG power BIS/RI-RASS	52	54	79	47	34	11	6	1
	Low ARV BIS/RI-RASS	36	54	57	20	8	2	2	-
	High ARV BIS/RI-RASS	55	31	33	24	25	6	4	1
Table 4	Stimulus–response	141	162	98	60	63**

### Analyses of variables

**Fig. 4:**
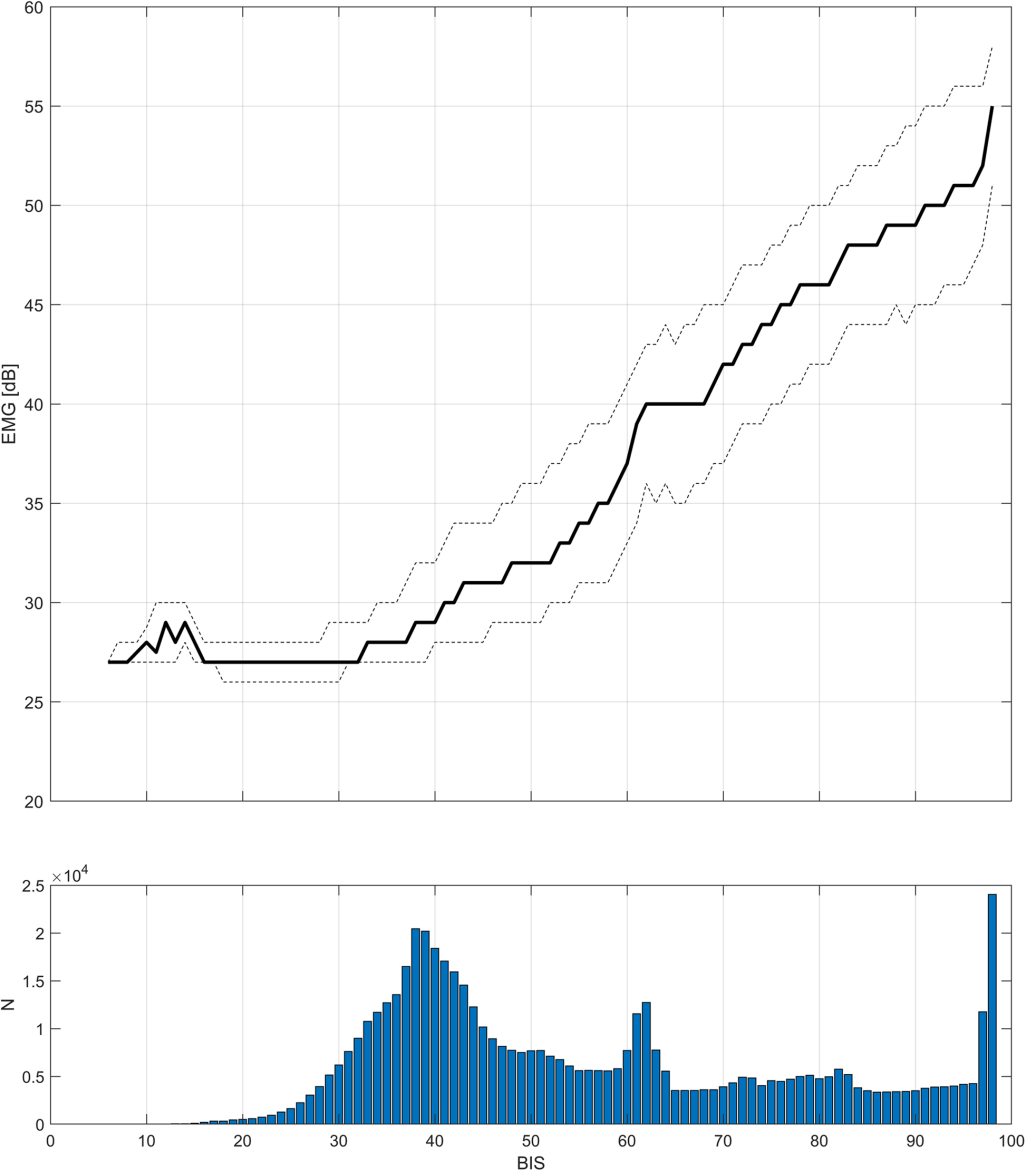
Top: Median (black line) and quartiles (grey dotted line) of EMG power, as a function of BIS using all available data of 30 patients. A linear correlation between EMG power and BIS is noted in the 30 to 50 dB EMG range. Bottom: A histogram of BIS values, using all available data from the 30 patients. The distribution of BIS is trimodal, possibly indicating switches between different BIS subparameters.

The correlation of EMG power versus BIS is presented in the top graph of Fig. 4, showing almost linear correlation in the BIS range of 40–95. At low EMG activity (EMG Power < 30 dB), the correlation with BIS is lost. The bottom graph of Fig. 4 presents the distribution of BIS values, showing that BIS values around 38, 62, and 98 are the most common.

**Fig. 5:**
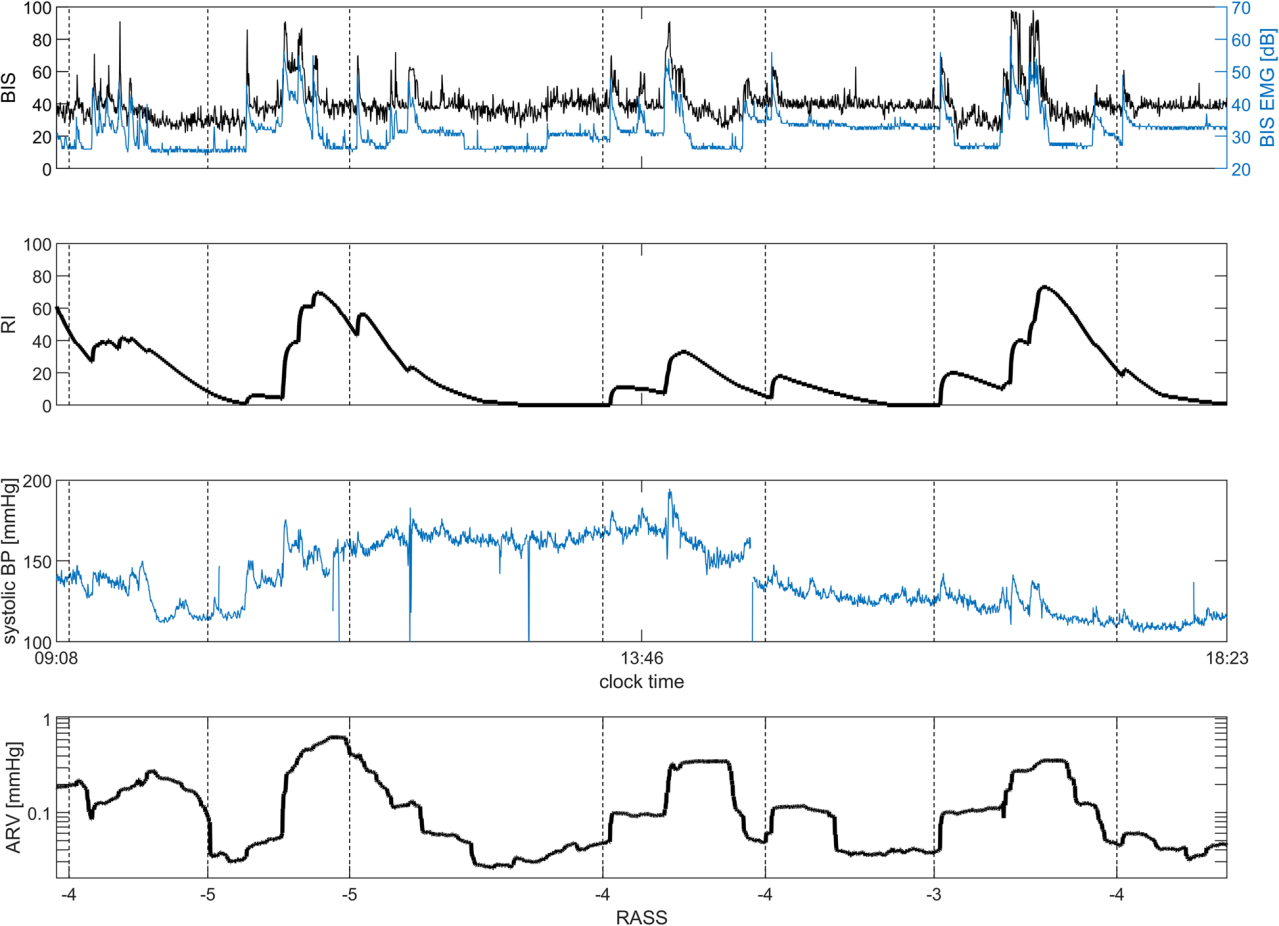
A monitoring example of a single study patient, presenting the continuous values of BIS EMG power (dB), Responsiveness Index (RI), systolic blood pressure [mmHg], blood pressure variability (ARV, note the logarithmic scale), and the timings of RASS assessments with the corresponding RASS value (dotted lines). During the monitoring period, the patient was sedated with a continuous propofol infusion and fentanyl bolus medication

As an example of similar behaviour of RI and ARV, the continuous monitoring data of a single patient can be seen in Fig. 5, presenting BIS, EMG power, RI, systolic blood pressure and ARV.

## Discussion

This explorative study shows that several easily measurable continuous physiological variables reflect the sedation level of ICU patients, as determined by the RASS scale, and also respond to painful stimuli in sedated, mechanically ventilated patients who are unable to report pain.

Of the studied variables, EEG and EMG derived variables were associated with RASS levels, as was demonstrated by the moderate P_K_ values of BIS, RI and EMG power. The variability of blood pressure, represented by ARV, showed no association with RASS levels. Interestingly, the P_K_ value of BIS was not substantially better than the P_K_ value of EMG power provided by the BIS monitor. It is a known fact that frontal EMG activity contaminates BIS values [30], and past BIS algorithm improvements have focused on decreasing the impact of EMG to BIS [13, 16]. The distribution of BIS values in this material is trimodal (Fig. 4 bottom graph), peaking at 38, 62 and 98. The actual depth of sedation is likely an approximately linear continuum, without three distict states of higher probability. The reason for this characteristic trimodal presentation of BIS is explained by earlier observations, where BIS “freezes” just below or above the recommended range of 40 to 60 for surgical anesthesia [31]. The BIS algorithm is a weighted sum of three subparameters (BetaRatio, SunchFastSlow, and BSR/QUAZI), which are weighted differently depending on the level of anaesthesia. BetaRatio is weighed for light sedation, SynchFastSlow for surgical anaesthesia, and BSR/QUAZI for deep anaesthesia [32], with histogram peaks at BIS 38 and 62. This may be related to the algorithmic switches between the assigned weight of different BIS subparameters [32]. Based on our data these switches may be triggered by the EMG value, since there are clear discontinuity points in the BIS-EMG association around BIS values of 40 to 60. As the BIS range of 40 to 60 is the recommended range for surgical anaesthesia, it is possible that the algorithm intentionally retards transitions over the cut-off values. Although the origin of frontal EMG activity remains obscure [33], anesthesiologists have utilized frontal EMG responsiveness for a long time in connection with painful stimuli [16, 34], and in later studies frontal EMG variability was found to be good classifier between nociceptive and non-nociceptive events during elective, noncardiac surgery with possible predictive power for movement responses [35].

Our results confirm the earlier findings of a correlation between BIS and frontal EMG in the ICU setting [29, 36], but contrary to earlier studies we demonstrated that this correlation could be a favorable property for BIS, as it seems to explain part of the association between BIS and RASS (Fig. 1). Riker and co-workers demonstrated a decreased correlation between BIS (version 3.2) and the Sedation-Agitation Scale (SAS) and the Visual Analog Scale (VAS), when EMG power was over 39 dB [36]. Tonner and co-workers compared one of the older BIS algorithms to a XP-level algorithm, demonstrating improved discrimination of Ramsay score levels with the XP-level system [29]. Our results confirm the results of Tonner and colleagues [29], who demonstrated enhanced discrimination between different sedation levels when EMG activity is over 30 dB (Kendall τ = -0.38 vs. τ = -0.26 for BIS XP).

Our study suggests that the RI reflects autonomous nervous system responses, and its reasonable capability to detect deep sedation is partly explained by the fact that sedative drugs attenuate those responses. Figure 2 reveals that RI is most often either 0 or 100, where the value 0 is more probable at RASS levels from -5 to -3, while the value 100 is more probable at RASS levels higher than -3. Moreover, the RI value of 100 tends to be less likely in the low ARV group. Thus, RI had difficulties in detecting light sedation (RASS levels -2 or higher) in calm patients with little or no blood pressure fluctuation. Low RI values in arousable patients have been reported earlier by Walsh et al., and were explained to be caused by sleep or minimal clinical stimulation [18]. Our data do not support the sleep hypothesis, as a majority of the concurrent BIS values were over 70 (Fig. 1), whereas the BIS values during sleep are typically less [37]. Figure 5 provides an example of a visual similarity of the RI and ARV trends, which for part supports our hypothesis that RI is linked to the effects of the autonomous nervous system. A recent study by Wennervirta et al*.* demonstrated a significantly higher incidence of hypertension (systolic blood pressure over 160 mmHg) in critical care patients when sedation was targeted to a RI level of 40 to 80, when compared to patients with a sedation target of RASS -3 to 0. This finding supports the hypothesis that RI mostly reflects sympathetic activity and has thus very limited applications in sedation titration [38].

Frontal EMG was the last remaining response to painful stimuli in the deepest sedation level (RASS scale -5). BIS was also reactive in RASS levels -4 and -3, but we assume that these responses are mainly explained by the EMG activation. Nociception monitors during surgical anaesthesia exists, but their application to critical care has been limited [8]. To our knowledge, this was the first preliminary study where the utility of SPI was assessed in the ICU setting. The capability of SPI to detect painful stimuli in ICU patients seems to be limited, and mostly explained, by the RRI response. It is, however, important to note that factors inherently affecting SPI were not excluded or controlled in this study, for instance atrial fibrillation, beta blockers, and pacemaker rhythm.

While HVR might be a potential tool for sedation-nociception monitoring, our data contained 19% of non-sinus rhythms, amount so high that it has a significant effect on the variable. In this study the finding was not expected, as previous studies do not report patient rhythm status. Another study of heart rate variability with only sinus rhythm might be interesting, however atrial arrhytmias and pacemaker rhythms are common in critical care patients.

The study results are limited by the explorative nature of the study, and by the small sample size. All results should be treated as hypothesis-generating and need to be validated with further research. The RASS assessment followed a standardized protocol (see Methods), but with two research nurses some inter-rater variability is bound to remain. Also, the results of the parameters responsiveness to painful stimuli should be taken as a preliminary finding, and these shall be confirmed in a future study with stricter study protocol and standardized stimuli. All the tested methods have specific limitations, which affect their potential use as monitoring methods. Apart from disconnection of monitoring devices, EEG is especially affected by artefacts (electrical disturbances) and drugs used for sedation and nociception. Similarly, NMBAs affect EMG, although frontal EMG is resistant to all but very deep relaxation. Sedation drugs, such as propofol, have hemodynamic effects (hypotension, reflectory tachycardia), although ARV should be reasonably resistant to a stable drug infusion. Assessing raw EEG and EMG data is challenging, and requires years of experience. On the other hand, computerized assessment of derived variables such as were studied in this study could overcome this challenge. The administration of sedation and analgesia was not standardised and varied both in dose and type of medication. While this represents the typical clinical ICU care, the results are affected by sedation and analgesia. Finally, data analysis was unfortunately delayed significantly, and while the results are still applicable, changes in critical care need to be considered when assessing the results of this study. Similarly, newer statistical methods and artificial intelligence methods might have provided more exact results, but were not available when the study data were collected.

## Conclusion

Variables derived from EEG (BIS) and EMG (EMG Power, RI) are useful for non-invasive nociception-sedation monitoring in mechanically ventilated ICU patients. Previously EMG has been considered as a disrupting artefact for derived EEG parameters, but our results show that EMG might be used as a part of monitoring in the ICU, where NMBAs are not typically used. EMG power can be useful for detecting responses to painful stimulation in critical care patients who are unable to communicate. As the individual response of each physiological variable to nociceptive stimulus was dependent on the RASS level, a multimodal approach including several of these variables could be beneficial in evaluating the level and adequacy of analgesia.

## Data Availability

All data are available freely by request from the corresponding author.

## Abbreviations

ARV: Averaged blood pressure variability
BIS: Bispectral index
BMI: Body mass index
BPV: Blood pressure variability
diaBP: Diastolic blood pressure
ECG: Electrocardiogram
EEG: Electroencephalogram
EMG: Electromyogram
ICU: Intensive care unit
LOS: Length of stay
NMBAs: Neuromuscular blockage agents
P_K_: Prediction probability
PPGA: Pulse photoplethysmographic waveform amplitude
RASS: Richmond-Agitation Sedation score
RI: Responsiveness Index
RRI: RR interval
SOFA: Sequential organ failure assessment
SPI: Surgical Pleth Index
sysBP: Systolic blood pressure
